# The effect of caffeine on cognitive performance is influenced by CYP1A2 but not ADORA2A genotype, yet neither genotype affects exercise performance in healthy adults

**DOI:** 10.1007/s00421-020-04384-8

**Published:** 2020-04-30

**Authors:** Alexander T. Carswell, Kevin Howland, Borja Martinez-Gonzalez, Pauline Baron, Glen Davison

**Affiliations:** 1 Endurance Research Group, School of Sport and Exercise Sciences, University of Kent, Chatham, Kent UK; 2 School of Biosciences, University of Kent, Canterbury, Kent UK

**Keywords:** Endurance exercise, Cognitive performance, Caffeine, Ergogenic, Genetics, Polymorphism

## Abstract

**Purpose:**

To determine the influence of two commonly occurring genetic polymorphisms on exercise, cognitive performance, and caffeine metabolism, after caffeine ingestion.

**Methods:**

Eighteen adults received caffeine or placebo (3 mg kg^−1^) in a randomised crossover study, with measures of endurance exercise (15-min cycling time trial; 70-min post-supplementation) and cognitive performance (psychomotor vigilance test; PVT; pre, 50 and 95-min post-supplementation). Serum caffeine and paraxanthine were measured (pre, 30 and 120-min post-supplementation), and polymorphisms in ADORA2A (rs5751876) and CYP1A2 (rs762551) genes analysed.

**Results:**

Caffeine enhanced exercise performance (*P* < 0.001), but effects were not different between participants with ADORA2A ‘high’ (*n* = 11) vs. ‘low’ (*n* = 7) sensitivity genotype (+ 6.4 ± 5.8 vs. + 8.2 ± 6.8%), or CYP1A2 ‘fast’ (*n* = 10) vs. ‘slow’ (*n* = 8) metabolism genotype (+ 7.2 ± 5.9 vs. + 7.0 ± 6.7%, *P* > 0.05). Caffeine enhanced PVT performance (*P* < 0.01). The effect of caffeine was greater for CYP1A2 ‘fast’ vs. ‘slow’ metabolisers for reaction time during exercise (− 18 ± 9 vs. − 1.0 ± 11 ms); fastest 10% reaction time at rest (− 18 ± 11 vs. − 3 ± 15 ms) and lapses at rest (− 3.8 ± 2.7 vs. + 0.4 ± 0.9) (*P* < 0.05). There were no PVT differences between ADORA2A genotypes (*P* > 0.05). Serum caffeine and paraxanthine responses were not different between genotypes (*P* > 0.05).

**Conclusion:**

Caffeine enhanced CYP1A2 ‘fast’ metabolisers’ cognitive performance more than ‘slow’ metabolisers. No other between-genotype differences emerged for the effect of caffeine on exercise or cognitive performance, or metabolism.

**Electronic supplementary material:**

The online version of this article (10.1007/s00421-020-04384-8) contains supplementary material, which is available to authorized users.

## Introduction

Caffeine is used globally by shift workers, military personnel, athletes, and others who need to overcome fatigue or prolong their capacity to complete occupational activities (Burke 2008). The ergogenic properties of caffeine were first reported more than a century ago (Rivers and Webber 1907), with its effects in reducing fatigue and enhancing wakefulness now well described (McLellan et al. 2016). Despite generally beneficial effects of caffeine on exercise and cognitive performance, sizeable inter-individual variations have been reported, including an absence of a positive effect in some individuals (Ganio et al. 2009; Grgic et al. 2018, 2019; Jenkins et al. 2008; McLellan et al. 2016; Southward et al. 2018). The efficacy of caffeine supplementation is affected by the dose, method and timing of ingestion, training status, and the performance measures examined (Burke 2008; McLellan et al. 2016). Evidence suggests genetic polymorphisms also influence an individual’s response to caffeine (Cornelis et al. 2006; Palatini et al. 2009; Retey et al. 2007). Mechanistically, caffeine is a potent adenosine receptor antagonist—caffeine blocks the actions of adenosine in the central nervous system, thereby decreasing feelings of tiredness and enhancing arousal, vigilance, and willingness to exert effort during exercise (Meeusen et al. 2013). The rs5751876 single-nucleotide polymorphism (SNP) in the ADORA2A gene which encodes for the adenosine A2A receptor has been used to categorise individuals as having a ‘high’ (TT genotype) or ‘low’ (CT or CC genotype) sensitivity to caffeine, and may account, in part, for some of this variability (Nehlig 2018). The P450 enzyme is responsible for 95% of the body’s caffeine metabolism—converting caffeine to its major metabolite paraxanthine—hence a polymorphism in the gene which encodes for the CYP1A2 isoform may also be responsible for some inter-individual differences. The rs762551 SNP can affect CYP1A2 enzyme activity and has been used to identify individuals as ‘fast’ (AA genotype) or ‘slow’ (AC or CC genotype) caffeine metabolisers (Nehlig 2018).

Only one study has examined the impact of ADORA2A genotype on the efficacy of caffeine in enhancing exercise performance, and it studied women only (Loy et al. 2015). Caffeine was found to improve 10-min time trial cycling performance in all participants with ‘high’ sensitivity, but in only one participant with ‘low’ sensitivity to caffeine. The largest randomised-controlled trial to examine CYP1A2 genotype and exercise performance found caffeine enhanced 10-km time trial cycling performance among only ‘fast’ caffeine metabolisers (Guest et al. 2018). Similarly, ‘fast’ metabolisers demonstrated a greater improvement in 40-km time trial performance following caffeine ingestion than ‘slow’ metabolisers (Womack et al. 2012). In contrast, caffeine increased power output to a greater extent in ‘slow’ compared with ‘fast’ metabolisers in a later study by the same laboratory (Pataky et al. 2016); but no effect of genotype was observed in their more recent study (Giersch et al. 2018), with both of these studies using a 3-km time trial to assess performance. As such, evidence to support an influence of CYP1A2 genotype on exercise performance is equivocal. Sleep-deprived individuals with ADORA2A ‘high’ sensitivity were found to have faster response times in a test of sustained attention after caffeine consumption than those with ‘low’ sensitivity (Bodenmann et al. 2012). Whether differences persist without sleep deprivation remains unclear. For example, caffeine was ergogenic for individuals with ‘high’ but not ‘low’ sensitivity to caffeine for one measure of reaction time, and vice versa for another (Renda et al. 2015). In the only study to date, CYP1A2 genotype did not influence visual attention test performance; however, the authors unexpectedly found that caffeine was not ergogenic for cognitive performance (Salinero et al. 2017). Interpreting the existing literature is further complicated by the fact that all but one of the aforementioned studies did not analyse the effect of genotype on caffeine metabolism (Giersch et al. 2018), to determine if/how this relates to any ergogenic differences.

The influence of genetic differences on the ergogenic effects of caffeine remains to be fully elucidated. To date, no single study has investigated the effect of both ADORA2A and CYP1A2 SNPs on exercise, cognitive performance or caffeine metabolism. Examining the influence of two commonly occurring genetic polymorphisms will give greater insight into potential mechanisms responsible for inter-individual variability. It has been suggested that these SNPs can provide further understanding of how an individual is likely to respond to caffeine and has the potential to underpin a move from a one-size-fits-all, to a personalised approach to the use of this popular ergogenic aid (Pickering and Kiely 2018). Therefore, the objective of this study was to determine the influence of ADORA2A and CYP1A2 SNPs on exercise, cognitive performance, and caffeine metabolism, after caffeine ingestion. It was hypothesised that the beneficial effects of caffeine would be greatest in individuals with ‘high’ sensitivity to caffeine (ADORA2A, TT genotype) and ‘fast’ caffeine metabolism (CYP1A2, AA genotype).

## Methods

### Participants

Eighteen young, healthy, active adults voluntarily gave written informed consent to participate in the study after being fully informed of the study procedures, verbally and in writing (age 24 ± 4 years; *n* = 12 men: body mass 74.7 ± 7.0 kg; height 1.78 ± 0.08 m; maximal oxygen uptake ($\dot{V}{\text{O}}_{{{\text{2max}}}}$) 49.5 ± 7.7 mL kg^−1^ min^−1^; *n* = 6 women: body mass 62.7 ± 10.4 kg; height 1.69 ± 0.08 m; $\dot{V}{\text{O}}_{{{\text{2max}}}}$ 43.2 ± 10.6 mL kg^−1^ min^−1^). All participants were free from any known immune, cardiovascular or metabolic diseases; were free from injury or illness; and were not taking any medication. Participants’ habitual daily caffeine intake was estimated using a food-frequency questionnaire and the typical caffeine content of consumed food, drink, and supplements (Burke 2008; Fitt et al. 2013; McLellan et al. 2016). Using previously defined thresholds (Womack et al. 2012), 13 participants were categorised as having a low caffeine intake (0–150 mg day^−1^); two participants had a moderate intake (151–300 mg day^−1^); and 3 participants had a high intake (> 300 mg day^−1^). The study received ethical approval from the University of Kent, School of Sport and Exercise Sciences Research Ethics and Advisory Group, and was conducted in accordance with the Declaration of Helsinki (2013).

### Study design

Using a double-blind, placebo-controlled crossover design, the effects of caffeine on endurance exercise performance, cognitive performance, and serum caffeine and paraxanthine were investigated. Caffeine and placebo trials were undertaken by participants in a randomised order, separated by 3–9 days, and a minimum of 2 days after completing preliminary measures. Using genomic DNA extracted from whole blood samples, SNPs in ADORA2A (rs5751876) and CYP1A2 (rs762551) genes were analysed, and the ergogenic effects of caffeine were compared between participants, categorised according to their genotype.

### Preliminary measures and familiarisation

Anthropometric measures were recorded on arrival at the laboratory. Following this, $\dot{V}{\text{O}}_{{{\text{2max}}}}$ was measured during a step incremental exercise test on an electromagnetically braked cycle ergometer (Excalibor Sport, Lode, Groningen, The Netherlands). The step incremental exercise test began at a power output of 100 W, increasing by 25 W every 3 min up to 200 W, following which the power output increased by 25 W every minute until participants reached volitional exhaustion. Pulmonary gas exchange was measured breath-by-breath for the duration of the test (Metalyser 3B, Cortex Biophysik, Leipzig, Germany). From this, the power output which elicited 70% $\dot{V}{\text{O}}_{{{\text{2max}}}}$ was calculated by interpolation of the power output–$\dot{V}{\text{O}}_{{{\text{2max}}}}$ relationship (using data from the submaximal stages). Maximal test workload (*W*_max_) was determined using the formula: *W*_max_ = power of the last fully completed stage + (*t*/60)·25 W where *t* was the duration (in seconds) of the final stage (Jeukendrup et al. 1997). For example, if a participant reached volitional exhaustion after 24 s of the 325 W stage, their *W*_max_ = 310 W. After 30-min rest, participants performed a familiarisation trial to verify the power output prescription of 70% $\dot{V}{\text{O}}_{{{\text{2max}}}}$ and accustom themselves to all trial procedures and tasks. Participants cycled for 20 min at the power output estimated to elicit 70% $\dot{V}{\text{O}}_{{{\text{2max}}}}$. After 5-min rest, participants then completed a 15-min cycling time trial during which they were instructed to perform as much work as possible. The ergometer was set in the linear mode, whereby the work rate increased linearly as a function of pedalling rate (squared). Linear mode was set according to the formula: *L* = 0.8·*W*_max_/(rpm)^2^ where *L* was the linear factor, *W*_max_ the maximal workload (from the step incremental test), and rpm the pedalling rate. Eighty percent of *W*_max_ was chosen following pilot testing, whereby the relative workload used by Jeukendrup et al. (1997) was increased to account for the shorter duration time trial used in the present study. The pedalling rate was determined from each participant’s average cadence during the step incremental exercise test. Participants were familiarised to the 10-min psychomotor vigilance test (PVT) by completing the test three times: twice whilst at rest and once during the 20 min of cycling at 70% $\dot{V}{\text{O}}_{{{\text{2max}}}}$. Three familiarisations were deemed sufficient to mitigate against learning effects (Lim and Dinges 2008).

### Experimental trials

**Fig. 1:**
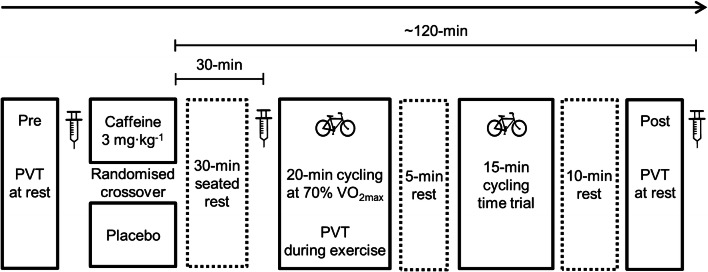
Schematic of experimental procedures. *PVT* psychomotor vigilance test; Syringe icon represents venous blood sample collected pre-supplementation, and 30 min and ~ 120 min post-supplementation

Participants reported to the laboratory after an overnight fast, and having avoided the consumption of caffeine-containing drinks, foods, and supplements for 48 h to control for any effect their habitual caffeine intake might have on the ergogenic effects of caffeine; and strenuous exercise for 24 h. Both caffeine and placebo trials were completed at the same time of day. Participants were given diet diaries to record all food and beverages consumed during the 24 h before their first experimental trial and instructed to replicate this before their second trial. On arrival at the laboratory, participants completed a pre-supplementation PVT whilst at rest and then provided a venous blood sample (Fig. 1). Participants then consumed a capsule containing 3-mg kg^−1^ body mass of caffeine (Food Grade; Sigma-Aldrich, Missouri, USA) during the caffeine trial, and an identical-looking capsule containing 3-mg kg^−1^ body mass of microcrystalline cellulose during the placebo trial. The capsule was swallowed with water proportional to their body mass (3-mL kg^−1^ body mass). During a subsequent 30 min of seated rest, participants consumed water ab libitum in their first trial and an equal volume in their second trial. Participants then provided a second blood sample. Forty-five minutes after consuming the caffeine or placebo capsule, participants commenced 20 min of cycling at the power output to elicit 70% $\dot{V}{\text{O}}_{{{\text{2max}}}}$. Participants completed a PVT during minutes 6–15, whilst continuing to exercise. After 5-min rest, during which participants consumed water (2 mL kg^−1^ body mass), participants completed a 15-min cycling time trial. Following 10-min rest, participants completed a PVT whilst at rest, and provided a final venous blood sample, ~ 120-min post-supplementation (118.5 ± 4.9 min). Participants wore a heart rate monitor during cycling, with heart rate recorded every minute (Polar Electro, Kempele, Finland). For the first 11 participants, rating of perceived exertion (RPE) was not recorded to minimise any interference and interaction with participants that might affect the primary study outcomes. After completing these trials, it was apparent that recording RPE using the Borg Scale during cycling at 70% $\dot{V}{\text{O}}_{{{\text{2max}}}}$ would be acceptable (during the 5-min periods before and after the PVT), so this measure was included for the final seven participants. All PVTs and cycling were completed in an environmental chamber (temperature 18.4 ± 1.0 °C; relative humidity 49.0 ± 7.5%).

### Endurance exercise performance

Participants were instructed to perform as much work as possible during the 15-min cycling time trial. All participants were briefed using the same standardised instructions before commencing cycling. The environmental chamber was silent and the only information which the participant received was the time remaining displayed on a digital timer in front of them. No fluids were consumed during the 15-min time trial. The ergometer was connected to a computer which calculated the total work performed, and was reported relative to their body mass.

### Cognitive performance

The 10-min PVT was completed on a laptop computer as a test of participants’ sustained attention (E-Prime, Psychology Software Tools, Sharpsburg, Pennsylvania, USA; Lim and Dinges 2008). Participants were instructed to focus on the screen for the whole test duration and press a response button as soon as a target appeared in the centre of the computer screen, which stopped the counter and displayed the reaction time for a 1-s period. The inter-stimulus interval, defined as the period between the last response and the appearance of the next stimulus, varied randomly from 2 to 10 s. Participants were instructed to press the button as soon as each stimulus appeared, to keep the reaction time as low as possible but not press the button too soon. Responses without a stimulus or with a reaction time < 100 ms were not counted. The primary PVT outcome measure was the mean reaction time. In addition, the following outcome metrics were analysed: mean fastest 10% reaction time; 1/reaction time (known as response speed); slowest 10% response speed; and number of lapses. Lapses were defined as a reaction time > 500 ms and were excluded from reaction time and response speed analyses (Lim and Dinges 2008). The PVT was conducted at rest whilst seated at a table, and during exercise on a cycle ergometer. During exercise, the keyboard was attached to the handlebars of the cycle ergometer. Participants wore their own spectacles during all PVTs as required. The environmental chamber was silent during all PVTs to minimise any distractions.

### Blood collection and handling

Whole blood samples were collected by venepuncture from an antecubital vein into one plain vacutainer tube and one K_2_EDTA tube (Becton Dickinson, Oxford, UK). An aliquot of whole blood was taken from the K_2_EDTA tube and refrigerated at 4 °C for up to 48 h before the extraction of DNA. Whole blood in plain vacutainer tubes was left to clot for 1 h. Subsequently, samples were centrifuged at 1500*g* for 10 min at 4 °C and serum aliquots stored at − 80 °C for later analysis.

### Serum caffeine and paraxanthine

Serum caffeine and paraxanthine concentrations were measured by high-performance liquid chromatography (HPLC) using a method modified from Holland et al. (1998). In preparation for analysis, serum samples were deproteinised by mixing 250 μL of thawed serum with 250 μL of 0.8-mol L^−1^ perchloric acid. Proteins were removed by centrifuging at 14,000*g* for 4 min at 18 °C. A 350-μL sample of supernatant was aliquoted into a 1.5-mL polypropylene microcentrifuge tube, and mixed with 27 μL of 4-mol L^−1^ sodium hydroxide, before being frozen at − 80 °C for later analysis. Deproteinised samples were defrosted and aliquoted into glass HPLC vials ready for analysis on an Agilent 1100 LC system fitted with a vacuum degasser, column oven, and diode array detector (Agilent Technologies, Santa Clara, California, USA). Caffeine and its metabolites were separated on a Phenomenex Synergi Hydro-RP column, 50 × 2.0 mm (2.5-μm particle size; Phenomenex, Macclesfield, UK). Deproteinised sample (5 μL) was injected, by autoinjector, and eluted isocratically over 10 min with 15 mmol L^−1^ potassium phosphate (pH 5.8):methanol (85:15, v/v) at a flow rate of 0.2 mL min^−1^ and column temperature of 30 °C. The column was flushed for 5 min with acetonitrile:water (80:20, v/v) and then re-equilibrated with the elution buffer for 10 min also at a flow rate of 0.2 mL min^−1^ and column temperature of 30 °C. Caffeine and its metabolites were detected by UV absorbance at 274 nm and peaks integrated with Agilent’s Chemstation software. Primary aqueous standards of caffeine and paraxanthine were prepared by dilution of a weighed sample of each substance (Sigma-Aldrich) into a defined volume of deionised water to give a concentration of 1 mg mL^−1^. An intermediate standard containing 100 μg mL^−1^ of both caffeine and paraxanthine was prepared by dilution of the primary standards in 5% bovine serum albumin (BSA; Sigma-Aldrich). The working standards for assay calibration (5, 2.5, 0.625, 0.156, 0.078, and 0.039 μg mL^−1^) were prepared by serial dilution of the intermediate standards in 5% BSA. Unspiked 5% BSA was used as a blank, and 0.625 μg mL^−1^ of caffeine and paraxanthine in 5% BSA used as a quality control, run after every 10 samples. A first tranche of analysis was completed for the first 11 participants. Data from our pilot study in 16 men and women also found no effect of genotype on caffeine metabolism 30- and 120-min post-caffeine ingestion (3 mg kg^−1^ body mass; Online Resource Supplementary Fig. 1); hence, it was deemed unnecessary to analyse serum caffeine and paraxanthine in the final seven participants.

### ADORA2A and CYP1A2 genotype

Genomic DNA was isolated from whole blood using the Zymo Miniprep Kit (Quick-DNA Miniprep Plus Kit, Zymo Research, Irvine, California, USA) and immediately frozen at − 80 °C for later analysis. Genotyping of the rs5751876 and rs762551 SNPs in the ADORA2A and CYP1A2 genes, respectively, was completed using rhAmp assays (Integrated DNA Technologies, Coralville, Iowa, USA). For the ADORA2A gene, participants were categorised as either TT homozygotes (‘high’ sensitivity) or carriers of the C allele (i.e., CT heterozygotes and CC homozygotes; ‘low’ sensitivity). For the CYP1A2 gene, participants were categorised as either AA homozygotes (‘fast’ metabolisers) or carriers of the C allele (i.e., AC heterozygotes and CC homozygotes; ‘slow’ metabolisers). Genotyping was conducted after participants had completed their experimental trials.

### Statistical analysis

A sample size estimation was calculated using the effect size reported by Loy and colleagues’ pilot study which used a 10-min time trial to assess exercise performance (Loy et al. 2015): Cohen’s *d* = − 1.89 for the difference in the beneficial effect of caffeine between ADORA2A genotypes (‘high’ vs. ‘low’ sensitivity). With alpha level set at 0.05, and power set at 0.8, recruiting 12 participants was deemed appropriate to detect a statistically significant difference in endurance exercise performance between ADORA2A genotypes (G*Power, version 3.1.9.2). One participant’s PVT data were excluded from analyses, because their number of lapses during exercise was > 2.5 standard deviations from the mean (Lee et al. 2010). Their profile also suggested that they did not follow the PVT instructions (i.e., focus on the screen for the duration of the test) during exercise. All data were checked for normality and sphericity. Independent *t* tests were used to analyse between-genotype differences in participant characteristics. Paired *t* tests were used to analyse the effect of caffeine on endurance exercise performance, heart rate, and RPE. Two-way repeated-measures ANOVA was used to analyse PVT reaction time, and serum caffeine, paraxanthine, and the paraxanthine:caffeine ratio, with Greenhouse–Geisser correction applied to the degrees of freedom if necessary. Bonferroni-adjusted paired *t* test post hoc procedures were used to determine within-participant differences where appropriate. Paraxanthine:caffeine ratio was not normally distributed; thus, a square root transformation was used to correct its positive skew. Caffeine–placebo change scores were calculated for endurance exercise performance, heart rate, PVT metrics, and serum caffeine and paraxanthine; with independent *t* tests used to analyse between-genotype differences. Where differences emerged, Cohen’s *d* effect sizes were calculated for the difference between change scores, where Cohen’s *d* greater than 0.2, 0.5, and 0.8 represent small, medium, and large effects, respectively (Cohen 1988). Data are presented as mean ± SD. Statistical analyses were performed using SPSS Statistics 25.0 (IBM, Armonk, New York, USA). Statistical significance was accepted at *P* < 0.05.

## Results

### ADORA2A and CYP1A2 genotype

For the rs5751876 SNP in the ADORA2A gene, 11 participants were homozygous for the T allele (TT, i.e., ‘high’ sensitivity); 6 participants were homozygous for the C allele (CC, i.e., ‘low’ sensitivity); and 1 participant was heterozygous (CT, i.e., ‘low’ sensitivity). For the rs762551 SNP in the CYP1A2 gene, 10 participants were homozygous for the A allele (AA, i.e., ‘fast’ metabolisers); 7 participants were heterozygous carriers of the C allele (AC, i.e., ‘slow’ metabolisers); and 1 participant was homozygous for the C allele (CC, i.e., ‘slow’ metaboliser). Seven participants were categorised as ‘high’ sensitivity, ‘fast’ metabolisers (i.e., ADORA2A, AA, and CYP1A2, TT). Participant characteristics were not different between genotypes (*P* > 0.05, Table 1); except ‘low’ sensitivity participants were taller than ‘high’ sensitivity participants (*P* < 0.05).

**Table 1:** Participant characteristics

	ADORA2A		CYP1A2	
	‘High’	‘Low’	‘Fast’	‘Slow’
Male	7	5	7	5
Female	4	2	3	3
Age (years)	23 ± 4	24 ± 5	23 ± 3	25 ± 5
Body mass (kg)	67.8 ± 10.5	75.3 ± 7.1	68.1 ± 11.0	73.9 ± 7.7
Height (m)^#^	1.72 ± 0.10	1.80 ± 0.05	1.73 ± 0.11	1.78 ± 0.04
BMI (kg m^−2^)	22.8 ± 2.3	23.1 ± 1.6	22.6 ± 2.3	23.4 ± 1.6
$\dot{V}{\text{O}}_{{{\text{2max}}}}$ (mL kg^−1^ min^−1^)	46.8 ± 10.4	48.4 ± 6.8	48.5 ± 6.3	46.2 ± 11.9
*W*_max_ (W)	269 ± 81	293 ± 33	271 ± 53	287 ± 83
Caffeine intake (mg day^−1^)	143 ± 139	104 ± 126	121 ± 128	135 ± 145
[Low, moderate, high]	[7, 2, 2]	[6, 0, 1]	[7, 2, 1]	[6, 0, 2]

Participants are categorised according to ADORA2A (‘high’ or ‘low’ sensitivity) and CYP1A2 genotypes (‘fast’ or ‘slow’ metaboliser)

*BMI* body mass index, $\dot{V}{\text{O}}_{{{\text{2max}}}}$ maximal oxygen uptake, *W*_*max*_ maximal test workload. Low, moderate, and high caffeine intake: 0–150, 151–300, and > 300 mg day^−1^, respectively. Data are mean ± SD, and *n* for sex and caffeine intake categories

^#^*P* < 0.05, ‘high’ vs. ‘low’ sensitivity

### Endurance exercise performance

**Fig. 2:**
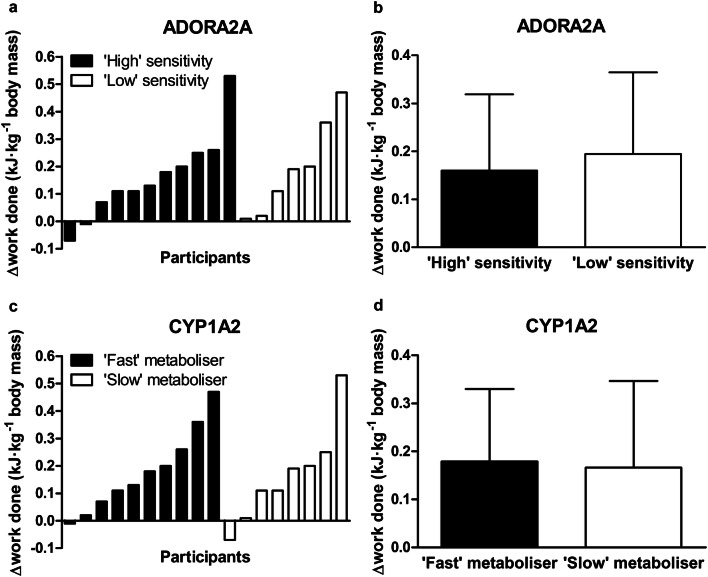
Caffeine–placebo change scores for total work performed during 15-min cycling time trial. **a**, **b** ADORA2A genotype ‘high’ (filled bars) and ‘low’ sensitivity to caffeine (open bars). **c**, **d** CYP1A2 genotype ‘fast’ (filled bars) and ‘slow’ caffeine metabolisers (open bars). **a**, **c** Bars represent individual participants. **b**, **d** Data are mean ± SD

Caffeine increased the total work completed during the 15-min cycling time trial by + 7.1 ± 6.1% compared with placebo (*P* < 0.001). The effect of caffeine on the total work completed was not different between ADORA2A genotypes (‘high’ sensitivity + 6.4 ± 5.8% vs. ‘low’ sensitivity + 8.2 ± 6.8%; *P* > 0.05, Fig. 2a, b). Similarly, the effect of caffeine on the total work completed was not different between CYP1A2 genotypes (‘fast’ metabolisers + 7.2 ± 5.9% vs. ‘slow’ metabolisers + 7.0 ± 6.7%; *P* > 0.05, Fig. 2c, d). Finally, the effect of caffeine on the total work completed was not different between ‘high’ sensitivity, ‘fast’ metabolisers compared with all others (‘high’ sensitivity, ‘fast’ metabolisers + 5.4 ± 3.4% vs. ‘others’ + 8.2 ± 7.2%; *P* > 0.05). Caffeine increased mean heart rate during the time trial compared with placebo (caffeine 179 ± 11 bpm vs. placebo 174 ± 12 bpm, *P* < 0.01), but not during 20 min of cycling at 70% $\dot{V}{\text{O}}_{{{\text{2max}}}}$ (caffeine 158 ± 13 bpm vs. placebo 158 ± 11 bpm, *P* > 0.05). The effect of caffeine on mean heart rate during the time trial was not different between ADORA2A genotypes (‘high’ sensitivity + 5 ± 5 bpm vs. ‘low’ sensitivity + 4 ± 5 bpm; *P* > 0.05); CYP1A2 genotypes (‘fast’ metabolisers + 5 ± 5 bpm vs. ‘slow’ metabolisers + 4 ± 6 bpm; *P* > 0.05); or between ‘high’ sensitivity, ‘fast’ metabolisers compared with all others (‘high’ sensitivity, ‘fast’ metabolisers + 5 ± 4 bpm vs. ‘others’ + 4 ± 6 bpm; *P* > 0.05). In the seven participants for whom RPE was measured during 20 min of cycling at 70% $\dot{V}{\text{O}}_{{{\text{2max}}}}$, there were no differences between caffeine and placebo at any time (minute 1: 11.6 ± 0.8 vs. 11.5 ± 1.0; minute 5: 13.3 ± 1.1 vs. 13.4 ± 0.9; minute 17: 14.4 ± 1.0 vs. 15.2 ± 1.5; minute 20: 14.9 ± 1.4 vs. 15.1 ± 1.6, all *P* > 0.05). No differences in RPE were apparent between genotypes.

### Cognitive performance

**Fig. 3:**
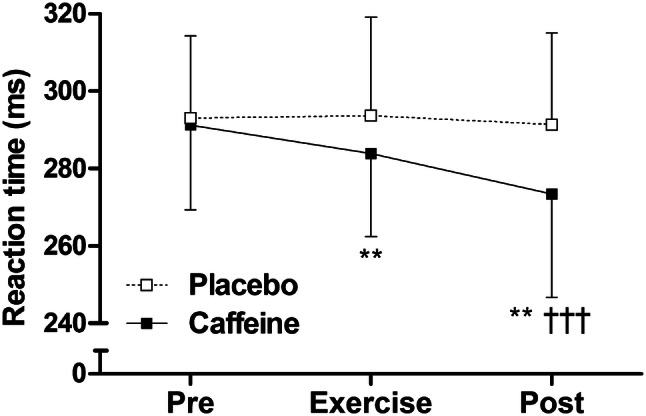
Psychomotor vigilance test reaction time for caffeine (filled squares) and placebo trials (open squares) measured at rest pre-supplementation, during exercise, and at rest post-supplementation. Data are mean ± SD. **P < 0.01, vs. placebo; ^†††^*P* < 0.001, vs. pre

**Fig. 4:**
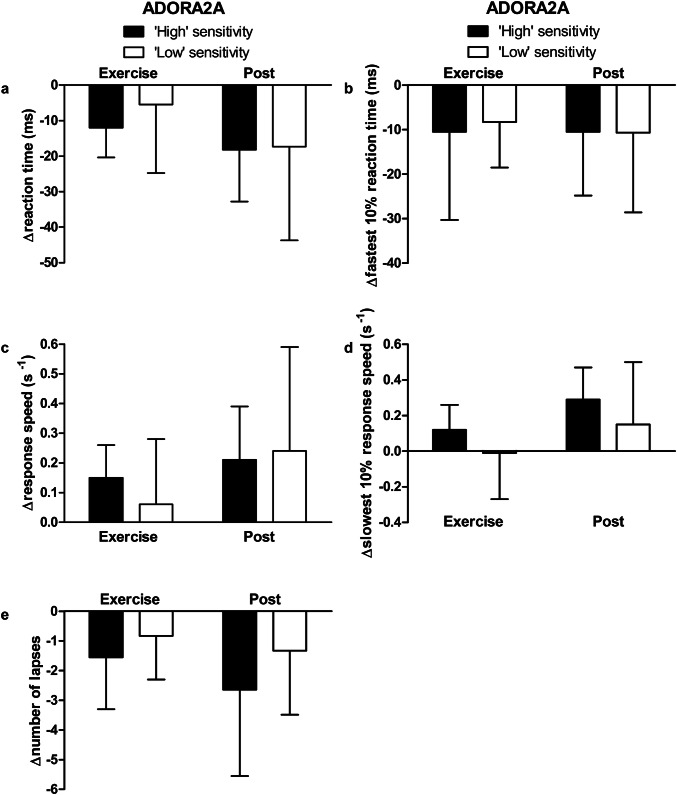
Psychomotor vigilance test caffeine–placebo change scores for **a** reaction time, **b** fastest 10% reaction time, **c** response speed, **d** slowest 10% response speed, and **e** number of lapses during exercise and at rest post-supplementation. Participants are categorised according to ADORA2A genotype: ‘high’ (filled bars) or ‘low’ sensitivity to caffeine (open bars). Data are mean ± SD

**Fig. 5:**
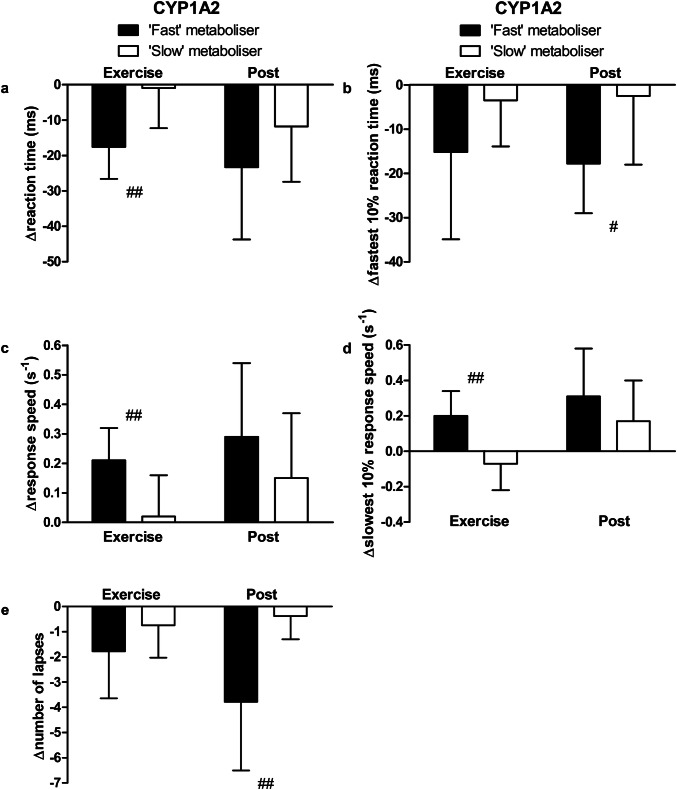
Psychomotor vigilance test caffeine–placebo change scores for **a** reaction time, **b** fastest 10% reaction time, **c** response speed, **d** slowest 10% response speed, and **e** number of lapses during exercise and at rest post-supplementation. Participants are categorised according to CYP1A2 genotype: ‘fast’ (filled bars) or ‘slow’ caffeine metabolisers (open bars). Data are mean ± SD. ^#^*P* < 0.05 and ^##^*P* < 0.01, between genotypes

Caffeine improved cognitive performance, with faster mean reaction times during exercise and at rest post-caffeine supplementation, compared with placebo (*P* < 0.01, Fig. 3). At rest pre-supplementation, there were no differences in caffeine–placebo PVT change scores between ADORA2A or CYP1A2 genotypes (*P* > 0.05). No differences in caffeine–placebo PVT change scores emerged between ADORA2A genotypes during exercise or at rest post-supplementation (‘high’ vs. ‘low’ sensitivity; *P* > 0.05, Fig. 4). During exercise, caffeine–placebo change scores for reaction time were lower in ‘fast’ compared with ‘slow’ metabolisers (*P* < 0.01, Cohen’s *d* = 1.6; Fig. 5a). Furthermore, caffeine–placebo change scores for response speed and slowest 10% response speed were higher among ‘fast’ compared with ‘slow’ metabolisers during exercise (*P* < 0.01, Cohen’s *d* = 1.5 and 1.9, respectively; Fig. 5c, d). In addition, caffeine–placebo change scores for the fastest 10% reaction time and number of lapses were lower for ‘fast’ compared with ‘slow’ metabolisers at rest post-supplementation (*P* < 0.05, Cohen’s *d* = 1.1 and *P* < 0.01, Cohen’s *d* = 1.7, respectively; Fig. 5b, e). No differences in PVT caffeine–placebo change scores emerged between ‘high’ sensitivity, ‘fast’ metabolisers compared with all others (*P* > 0.05).

### Serum caffeine and paraxanthine

**Fig. 6:**
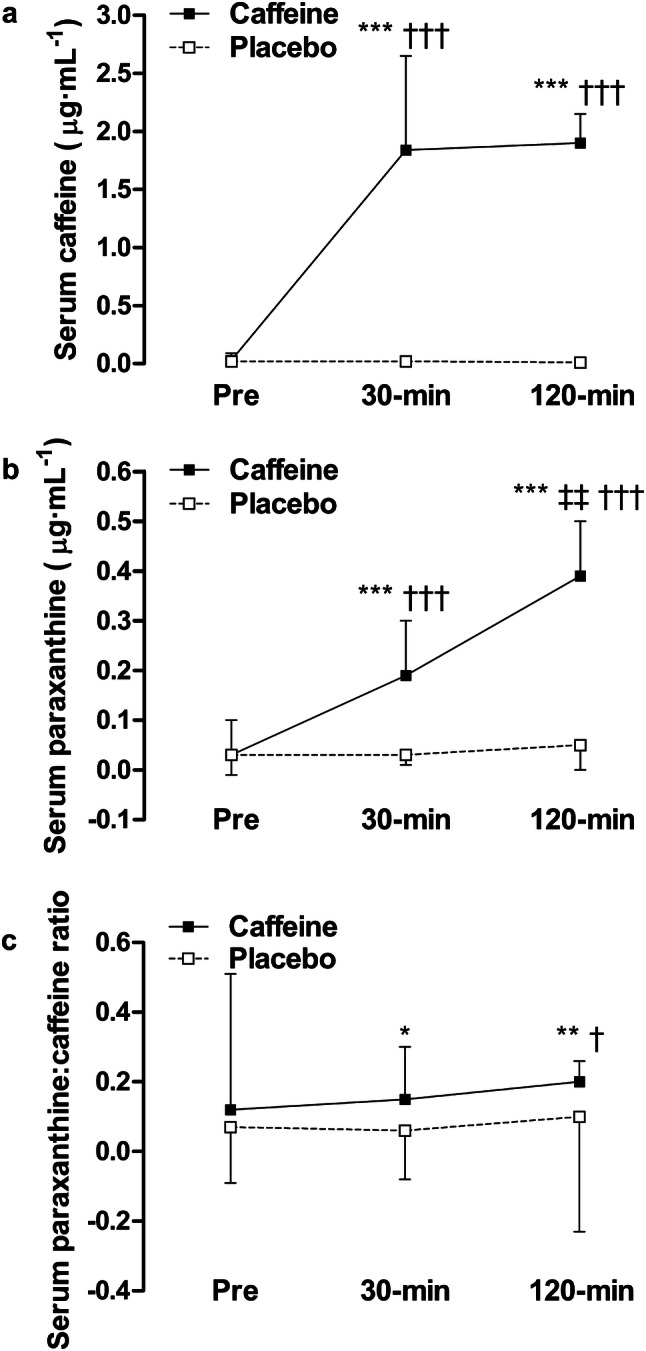
Serum **a** caffeine, **b** paraxanthine, and **c** the paraxanthine:caffeine ratio for caffeine and placebo trials measured pre-supplementation, and 30-min and ~ 120-min post-supplementation. Data are mean ± SD. *P < 0.05, **P < 0.01 and ***P < 0.001, vs. placebo; ^‡‡^*P* < 0.01, vs. 30 min; ^†^*P* < 0.05 and ^†††^*P* < 0.001, vs. pre

Serum caffeine and paraxanthine concentrations increased after caffeine ingestion, and were higher than during the placebo trial, 30 and ~ 120 min post-supplementation (*P* < 0.001, Fig. 6a, b). The serum paraxanthine:caffeine ratio increased after caffeine consumption, and was greater than during the placebo trial after 30 and ~ 120 min (*P* < 0.05, Fig. 6c). No differences in caffeine–placebo change scores for serum caffeine, paraxanthine, or the paraxanthine:caffeine ratio emerged between genotypes (‘high’ vs. ‘low’ sensitivity, ‘fast’ vs. ‘slow’ metabolisers, and ‘high’ sensitivity, ‘fast’ metabolisers vs. ‘others’; *P* > 0.05, Table 2).

**Table 2:** Caffeine–placebo change scores for serum caffeine, paraxanthine and the paraxanthine:caffeine ratio, 30 and ~ 120-min post-supplementation

	ADORA2A				CYP1A2			
	30 min		120 min		30 min		120 min	
	‘High’	‘Low’	‘High’	‘Low’	‘Fast’	‘Slow’	‘Fast’	‘Slow’
Δserum caffeine (μg mL^−1^)	+ 1.65 ± 0.95	+ 2.12 ± 0.33	+ 1.95 ± 0.26	+ 1.79 ± 0.20	+ 1.95 ± 0.84	+ 1.67 ± 0.80	+ 1.97 ± 0.28	+ 1.80 ± 0.16
Δserum paraxanthine (μg mL^−1^)	+ 0.13 ± 0.10	+ 0.21 ± 0.12	+ 0.32 ± 0.10	+ 0.36 ± 0.20	+ 0.18 ± 0.13	+ 0.15 ± 0.08	+ 0.31 ± 0.12	+ 0.37 ± 0.15
Δserum paraxanthine:caffeine ratio	+ 0.17 ± 0.18	− 0.04 ± 0.18	+ 0.19 ± 0.05	− 0.05 ± 0.57	+ 0.08 ± 0.26	+ 0.11 ± 0.14	+ 0.01 ± 0.44	+ 0.23 ± 0.06

Participants are categorised according to ADORA2A (‘high’ or ‘low’ sensitivity) and CYP1A2 genotypes (‘fast’ or ‘slow’ metaboliser)

Data are mean ± SD. *P* > 0.05, ‘high’ vs. ‘low’ sensitivity and ‘fast’ vs. ‘slow’ metaboliser

## Discussion

This randomised, placebo-controlled trial is the first to examine both the separate and combined influence of two genetic polymorphisms (ADORA2A rs5751876 and CYP1A2 rs762551) on the ergogenic effects of caffeine (3 mg kg^−1^ body mass). Aligned with the hypothesis, CYP1A2 genotype did affect cognitive performance—with a greater beneficial effect of caffeine observed in ‘fast’ metabolisers (Fig. 5). In contrast and contrary to the hypothesis, ADORA2A genotype did not influence the positive effect of caffeine on cognitive performance (Fig. 4). Neither ADORA2A nor CYP1A2 genotype affected the ergogenic effect of caffeine on endurance exercise performance (Fig. 2). With regards to caffeine metabolism, genotype did not affect serum caffeine or paraxanthine concentrations 30 and ~ 120-min post-caffeine consumption (Table 2). The effect of caffeine on exercise and cognitive performance and metabolism was not different between individuals with ‘high’ sensitivity, ‘fast’ metabolism, and all others.

### Endurance exercise performance

This is the first study to examine the effect of ADORA2A genotype on exercise performance in a cohort of men and women. The present results show that ADORA2A genotype did not influence performance in a 15-min cycling time trial. These novel findings contrast with the only existing study, where caffeine (5-mg kg^−1^ body mass) was ergogenic in a 10-min time trial for only ‘high’ sensitivity women (Loy et al. 2015). In the present study, all but one of the ‘low’ sensitivity participants were homozygous for the C allele. The inclusion of several ‘low’ sensitivity women heterozygous for the C allele by Loy et al. (2015) may account for these divergent findings—it remains unclear whether CT and CC individuals respond differently to caffeine. To illustrate, null and detrimental effects of caffeine on exercise performance have been seen in hetero- and homozygous CYP1A2 ‘slow’ metabolisers, respectively (Guest et al. 2018). ADORA2A genotype has been found to be associated with habitual caffeine intake (Cornelis et al. 2007), which could account for between-genotype differences in performance following acute supplementation (Bell and McLellan 2002). There were no between-genotype differences in habitual caffeine consumption in the present study—furthermore, in contrast to Bell and McLellan’s (2002) methodology, participants in the present study were instructed to abstain from caffeine for 48 h prior to the experimental trials to help control for any effect their habitual intake might have on the ergogenic effects of caffeine. This approach also allowed the effect of genotype per se to be examined, rather than a phenotypic response influenced by habitual intake. Future studies aiming to examine the potential influence of habitual caffeine intake on caffeine sensitivity would require larger numbers of individuals with low and high caffeine intake. The most ecologically valid design for this purpose would also require participants to continue with their normal habitual intake in the days before the experimental trials.

That CYP1A2 genotype did not affect exercise performance is in agreement with the previous studies that have assessed performance using a 15-min time trial (Algrain et al. 2016) and a shorter duration 3-km time trial (Giersch et al. 2018). However, no ergogenic effect of caffeine was observed in the 15-min time trial study of Algrain et al. (2016) (using caffeinated chewing gum)—probably precluding an effect of genotype. A caffeine dose > 3-mg kg^−1^ body mass may be necessary for genotypic differences to be detected, because in all studies where an influence of genotype on endurance exercise performance has emerged, a 4–6-mg kg^−1^ body mass dose was used (Guest et al. 2018; Loy et al. 2015; Pataky et al. 2016; Womack et al. 2012). However, this theory is not supported by Geirsch et al. (2018), where CYP1A2 genotype did not influence 3-km time trial performance despite 6-mg kg^−1^ body mass caffeine ingestion. It may be caffeine enhances ‘fast’ metabolisers’ performance more than ‘slow’ metabolisers only in performance tests of a duration greater than 15 min; for example, a 10- or 40-km time trial (Guest et al. 2018; Womack et al. 2012). This proposal is supported by the absence of an effect of genotype in the present study, and on Wingate power output (Salinero et al. 2017), a 3-km time trial (Giersch et al. 2018), and contradictory greater benefit of caffeine in ‘slow’ metabolisers in a 3-km time trial (Pataky et al. 2016). The relevance of the (i) caffeine dose and (ii) duration of exercise performance tests may be related to caffeine metabolism and between-genotype variability in the availability of caffeine and its metabolites. However, no measurements of caffeine metabolites were made in studies where genotype has been shown to influence exercise performance (Guest et al. 2018; Loy et al. 2015; Pataky et al. 2016; Womack et al. 2012), and hence, the role of metabolism per se remains unproven.

### Caffeine metabolism

There were no between-genotype differences in caffeine metabolism in the present study. In further support of these findings, data from our pilot study in healthy men and women (*n* = 16) also found no effect of genotype on caffeine metabolism 30 and 120-min post-caffeine ingestion (3-mg kg^−1^ body mass; Online Resource Supplementary Fig. 1). Similarly, an earlier study found that caffeine pharmacokinetics were not different between ‘fast’ and ‘slow’ metabolisers up to 65 min after chewing gum containing 300 mg of caffeine (Algrain et al. 2016). The absence of an effect of CYP1A2 genotype on exercise performance in the present study is possibly due to the observed lack of any pharmacokinetic differences over the study period (i.e., up to 120 min after caffeine ingestion). Faster clearance of caffeine, as was anticipated to occur in ‘fast’ metabolisers, may mean impairments in exercise performance relative to ‘slow’ metabolisers are avoided, because a build-up of caffeine can block adenosine-induced vasodilatation in the coronary arteries, thereby reducing myocardial blood flow during exercise (Higgins and Babu 2013). There were no between-genotype differences in heart rate in the present study, suggesting that myocardial blood flow was not impaired. Caffeine metabolites paraxanthine and theophylline are potent adenosine receptor antagonists, and may themselves be ergogenic (Graham 2001). Therefore, faster caffeine metabolism and subsequent higher metabolite concentrations may result in superior performance. For between-genotype differences in exercise performance to emerge, the concentration of caffeine metabolites, rather than caffeine alone, may need to be affected by genotype. For example, a previous randomised-controlled trial showed that despite reportedly higher serum caffeine concentrations in ‘slow’ metabolisers 1 h post-caffeine consumption (in contrast to the present results), no differences in paraxanthine, theobromine, theophylline, or exercise performance emerged between ‘fast’ and ‘slow’ metabolisers (Giersch et al. 2018).

The present study lends support to an absence of an effect of CYP1A2 genotype on caffeine metabolism 30- and ~ 120-min post-caffeine ingestion—the window during which most caffeine supplementation exercise studies assess performance. In studies that have found a performance difference between ‘fast’ and ‘slow’ metabolisers (Guest et al. 2018; Pataky et al. 2016; Womack et al. 2012), the absence of serum metabolite measures means that there is currently no evidence to indicate that differences in caffeine metabolism are responsible per se. In all of these studies, performance was completed within 120 min of supplementation, so the between-genotype differences observed were unlikely to have been caused by direct differences in caffeine metabolism. The influence of some other mechanism attributable to between-genotype differences remains a possibility. What other mechanism may be responsible (for example local tissue differences in metabolism) remains to be determined. Importantly, caffeine concentrations measured up to an hour after ingestion may be more reflective of caffeine absorption than metabolism (Graham 2001). It is possible that metabolic and performance differences will emerge between genotypes later post-supplementation. For example, metabolic activity (ratio of plasma paraxanthine and caffeine) has previously been shown to be higher in ‘fast’ compared with ‘slow’ metabolisers 5 h after the ingestion of 100 mg of caffeine (Sachse et al. 1999). No between-genotype differences in serum caffeine or paraxanthine were present ~ 2 h post-caffeine consumption in the present study. Future studies are required to investigate if any between-genotype differences in pharmacokinetics emerge over a longer time course of several hours (it is also interesting that the difference between habitual caffeine users and non-users was most apparent at 6-h post-supplementation in the study of Bell and McLellan (2002), given that genotype might influence habitual intake in some individuals). It follows that performance measures commencing later or continuing for longer after caffeine ingestion also warrant further investigation.

### Cognitive performance

ADORA2A genotype did not affect the positive effect of caffeine on cognitive performance. In contrast, caffeine enhanced CYP1A2 ‘fast’ metabolisers’ cognitive performance more than ‘slow’ metabolisers during exercise and at rest. In the only other study to date, no effect of CYP1A2 genotype on reaction times was observed; however, the absence of an ergogenic effect of caffeine likely precluded the detection of a caffeine–gene interaction (Salinero et al. 2017). Large effect sizes for the difference between ‘fast’ and ‘slow’ metabolisers’ PVT change scores were observed in the present study, suggesting that these differences may be of practical significance. A possible mechanism to explain the effects on cognitive performance in ‘fast’ metabolisers could be a greater availability of caffeine metabolites within the central nervous system where they are active and bind to the adenosine receptor, despite no between-genotype differences in serum. Indeed, the concentration of ‘free’ caffeine and paraxanthine in serum may not reflect the concentration bound to adenosine receptors, which may have been greater in ‘fast’ metabolisers.

### Strengths and limitations

The 18 participants in the present study were sufficient to meet the calculated sample size requirement, and the proportion of individuals with ‘high’ and ‘low’ sensitivity (61% and 39%, respectively) and ‘fast’ and ‘slow’ metabolism genotypes (56% and 44%, respectively) were similar to those previously reported (Nehlig 2018). However, this relatively small sample size meant that only one participant was a heterozygous carrier of the ADORA2A C allele, and only one participant was homozygous for the CYP1A2 C allele. Future studies are required to determine the influence of these specific genotypes on the ergogenic effects of caffeine. However, the present study’s finding that CYP1A2 genotype influences the effect of caffeine on cognitive performance has practical relevance to the majority of the population, since approximately 90% of individuals are CYP1A2 AA or AC carriers (Pickering and Kiely 2018). The findings of the present study among healthy men and women may not be applicable to athletes due to differences in fitness. A notable strength of the present study was cognitive performance was assessed during exercise and at rest using a PVT, which is a widely used test of sustained attention with excellent reliability and validity (Lim and Dinges 2008). The assessment of cognitive performance during exercise may be particularly relevant for athletes and others who engage in exercise and cognitive tasks simultaneously. Further strengths of the experimental approach used in the current study include the recruitment of both men and women; the use of an ecologically valid dose of caffeine and timing prior to performance assessments—typically used by athletes and others (Maughan et al. 2018; Meeusen et al. 2013); and the recording of heart rate during exercise, and the analysis of caffeine metabolism to better understand the potential mechanisms of action. Future studies are required to understand the possible role of other polymorphisms in ADORA2A, CYP1A2, and other genes involved in the response to or metabolism of caffeine before any personalised recommendations for the use of this ergogenic aid can be made based on genotype.

## Conclusions

The present study is the first to investigate the separate and combined influence of two SNPs (ADORA2A rs5751876 and CYP1A2 rs762551) on the ergogenic effects of caffeine. The beneficial effect of caffeine on cognitive performance during exercise and at rest was greater in CYP1A2 ‘fast’ metabolisers compared with ‘slow’. In contrast, there were no differences in cognitive performance between individuals with ADORA2A ‘high’ or ‘low’ sensitivity to caffeine. Furthermore, no differences in endurance exercise performance or caffeine metabolism emerged between ADORA2A or CYP1A2 genotypes. The influence of caffeine on metabolism, and exercise and cognitive performance was not different between ‘high’ sensitivity, ‘fast’ metabolisers and all other individuals. The present study provides evidence that an SNP in the CYP1A2 gene—central to caffeine metabolism—has a role in the inter-individual variability of cognitive performance enhancement observed after caffeine ingestion. It would be of interest to examine the influence of other polymorphisms in these genes and others involved in caffeine’s ergogenic actions in a larger sample that includes more ‘well-trained’ athletes.

## Electronic supplementary material

Below is the link to the electronic supplementary material.

Supplementary file1 (PDF 112 kb)

## Data Availability

Not applicable.

## Abbreviations

ANOVA: Analysis of variance
BMI: Body mass index
BSA: Bovine serum albumin
DNA: Deoxyribonucleic acid
HPLC: High-performance liquid chromatography
*L*: Linear factor
PVT: Psychomotor vigilance test
RPE: Rating of perceived exertion
rpm: Revolutions per minute
SD: Standard deviation
SNP: Single-nucleotide polymorphism
*t*: Time
$\dot{V}{\text{O}}_{{{\text{2max}}}}$: Maximal oxygen uptake
*W*_max_: Maximal test workload
