# Supplementary material for: The effect of caffeine on cognitive performance is influenced by CYP1A2 but not ADORA2A genotype, yet neither genotype affects exercise performance in healthy adults
Source: Eur J Appl Physiol. 2020 Apr 30;120(7):1495–508. doi: 10.1007/s00421-020-04384-8 (PMC7295849; doi:10.1007/s00421-020-04384-8)
Supplement: Supplementary file 1 — Supplementary file1 (PDF 112 kb) [file 421_2020_4384_MOESM1_ESM.pdf]

The effect of caffeine on cognitive performance is influenced by CYP1A2 but not ADORA2A genotype, yet neither genotype affects exercise performance in healthy adults

European Journal of Applied Physiology

Alexander T. Carswell[1], Kevin Howland[2], Borja Martinez-Gonzalez[1], Pauline Baron[1] and Glen Davison[1]

[1]Endurance Research Group, School of Sport and Exercise Sciences, University of Kent, Chatham, Kent, United Kingdom

[2]School of Biosciences, University of Kent, Canterbury, Kent, United Kingdom

Corresponding author: Dr. Glen Davison

Email address: g.davison@kent.ac.uk

Electronic supplementary material

Supplementary Fig. 1 Serum (a, b, c) caffeine, (d, e, f) paraxanthine, and (g, h, i) the paraxanthine:caffeine ratio measured pre-supplementation, and 30-min and 120-min post-caffeine supplementation (3 mg·kg^{-1} body mass). Participants (*n* = 16) are categorised according to ADORA2A genotype (a, d, g: 'high', *n* = 10 or 'low' sensitivity, *n* = 6); CYP1A2 genotype (b, e, h: 'fast', *n* = 8 or 'slow' metaboliser, *n* = 8); and ADORA2A and CYP1A2 genotypes (c, f, i: 'high' sensitivity, 'fast' metaboliser, *n* = 6 or 'others', *n* = 10). Data are mean ± SD. Mixed model ANOVA genotype x time interaction $P > 0.05$ for caffeine, paraxanthine and the paraxanthine:caffeine ratio

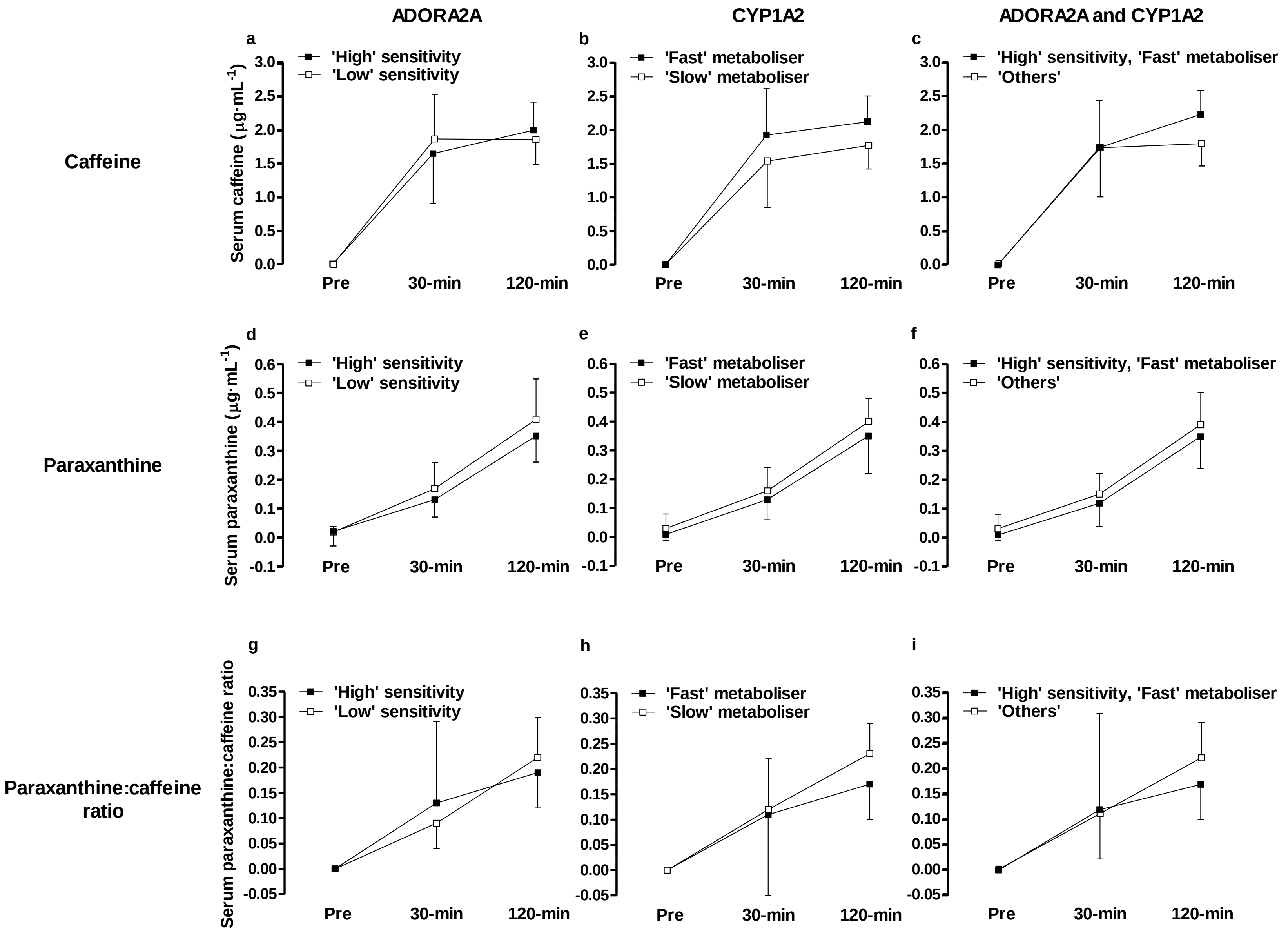

ADORA2A
CYP1A2
ADORA2A and CYP1A2
Caffeine
Paraxanthine
Paraxanthine:caffeine ratio
a
b
c
d
e
f
g
h
i
'High' sensitivity
'Low' sensitivity
'Fast' metaboliser
'Slow' metaboliser
'High' sensitivity, 'Fast' metaboliser
'Others'
Serum caffeine (µg·mL-1)
Serum paraxanthine (µg·mL-1)
Serum paraxanthine:caffeine ratio
Pre
30-min
120-min
0.0
0.5
1.0
1.5
2.0
2.5
3.0
-0.1
0.1
0.2
0.3
0.4
0.5
0.6
-0.05
0.00
0.05
0.10
0.15
0.20
0.25
0.30
0.35
